# Baseline Serum HE4 But Not Tissue HE4 Expression Predicts Response to the Levonorgestrel-Releasing Intrauterine System in Atypical Hyperplasia and Early Stage Endometrial Cancer

**DOI:** 10.3390/cancers12020276

**Published:** 2020-01-23

**Authors:** Roya Behrouzi, Neil A. J. Ryan, Chloe E. Barr, Abigail E. Derbyshire, Y. Louise Wan, Zoe Maskell, Katie Stocking, Philip W. Pemberton, James Bolton, Rhona J. McVey, Emma J. Crosbie

**Affiliations:** 1 Department of Medicine, Manchester University NHS Foundation Trust, Manchester M13 9WL, UK; rbehrouzi@doctors.org.uk; 2 Division of Cancer Sciences, Faculty of Biology, Medicine and Health, University of Manchester, St. Mary’s Hospital, Manchester M13 9WL, UK; neilryan@nhs.net (N.A.J.R.); Yee-loi.Wan@manchester.ac.uk (Y.L.W.); zoemaskell@googlemail.com (Z.M.); 3 Department of Obstetrics and Gynaecology, St. Mary’s Hospital, Manchester University NHS Foundation Trust, Manchester Academic Health Science Centre, Manchester M13 9WL, UK; Chloe.barr@mft.nhs.uk (C.E.B.); abiderbyshire@doctors.org.uk (A.E.D.); 4 Centre for Biostatistics, Division of Population Health, Health Services Research and Primary Care, University of Manchester, Manchester M13 9PL, UK; katie.stocking@manchester.ac.uk; 5 Department of Clinical Biochemistry, Manchester University NHS Foundation Trust, Manchester Academic Health Science Centre, Manchester M13 9WL, UK; philipwpemberton@hotmail.co.uk; 6 Department of Pathology, Manchester University NHS Foundation Trust, Manchester Academic Health Science Centre, Manchester M13 9WL, UK; James.bolton@mft.nhs.uk (J.B.); rhona.mcvey@mft.nhs.uk (R.J.M.)

**Keywords:** HE4, endometrial cancer, atypical hyperplasia, biomarker, therapy, response, levonorgestrel-releasing intrauterine system, LNG-IUS

## Abstract

The levonorgestrel-releasing intrauterine system (LNG-IUS) is a conservative management option for atypical hyperplasia (AH) and low grade early stage endometrial cancer (EEC), but around 1 in 3 patients fail to respond to treatment. The aim of this study was to investigate if serum and/or tissue HE4 expression could predict response to LNG-IUS therapy. Patients with AH or presumed Stage I EEC had serum and endometrial samples taken at baseline and at 3-month intervals over 12 months post-insertion of LNG-IUS. 74 patients were recruited and baseline demographics recorded. Of 57 patients for whom response was histologically determinable, 39 (68%) were responders and 18 (32%) non-responders. Mean baseline serum HE4 was significantly lower in responders (62.1 ± 1.1 pM, 95% confidence interval (CI) 52.7–73.2), compared to non-responders (125.6 ± 1.3 pM, 95% CI 74.5–211.7, *p* = 0.014), including when considering age, BMI, menopausal status, smoking status, and histological grade as covariables (*p* = 0.005). Baseline tissue HE4 expression was not significantly different in responders compared to non-responders (*p* = 0.999). Responders showed a significant mean reduction (−9.8 ± 3.4%, 95% CI −16.7 to −2.8%, *p* = 0.008) in serum HE4 between baseline and 3 months (*p* = 0.008), whereas non-responders showed no significant change (*p* = 0.676). Neither responders nor non-responders showed a significant percentage change in serum HE4 from baseline beyond 3 months (*p* > 0.05). Change in serum HE4 between baseline and 3 and 6 months and tissue HE4 tissue expression between baseline and 3, 6, and 12 months was not significantly different in responders compared to non-responders (*p* > 0.05). This study suggests that baseline serum HE4, but not baseline tissue HE4 expression, is independently predictive of response to the LNG-IUS and could be used to guide management decisions.

## 1. Introduction

Endometrial carcinoma (EC) is the most common gynaecological malignancy and frequently presents at an early stage. A total of 70–80% of ECs are endometrioid endometrial adenocarcinomas (EECs), and atypical endometrial hyperplasia (AH) is a precursor lesion that progresses to EEC in 8–29% of cases [1]. Hysterectomy is currently the first line treatment for AH and stage Ia EEC. Where surgery is contraindicated or fertility preservation desired, progestogen therapy is a non-surgical treatment option. Studies have shown that oral progestogens can reverse AH and Stage Ia EEC in 70–75% patients by 12 months [2,3,4,5,6]. Further studies have shown that the levonorgestrel-releasing intrauterine system (LNG-IUS) produces fewer systemic side effects and regression rates of 84–100% in AH [7,8,9,10,11] and 68% in stage I EEC [12]. This has led to increased use of the LNG-IUS compared to oral progestogens as a conservative therapy. There are currently no biomarkers available to predict or monitor response to treatment with the LNG-IUS [13] and reliance is on close surveillance with imaging and/or endometrial biopsies at regular intervals. With up to 25% of EEC patients being pre-menopausal [14], and the rising prevalence of obesity and ageing population, increasing numbers of patients are likely to require conservative management in the future. Since up to a third of patients do not respond to LNG-IUS treatment, there is a demand for biomarkers that could be used to predict and/or monitor therapy response.

Human epididymis protein 4 (HE4) has been recently identified as a potential biomarker in endometrial cancer [15]. It is a glycoprotein expressed in various human tissues, including normal epithelium of the female genital tract [16]. It is overexpressed in many cancers and detectable histologically in 90% of EECs [16]. Serum HE4 is also raised in patients with EEC and has been shown to have higher sensitivity than CA-125 for detection of EEC at both early and advanced stages [17,18,19,20]. Studies have shown that elevated serum HE4 correlates positively with advanced age, menopause, higher FIGO stage and grade, greater tumour size, deep myometrial invasion, lymph node involvement and reduced survival [17,20,21,22,23,24]. Higher tissue expression of HE4 has also been associated with higher histological grades, higher stages and increased mortality in AH and EEC [24,25]. This has sparked interest in HE4 as a potential tissue and serum biomarker for predicting and monitoring therapy response to progestogens. A recent study found that changes in HE4 tissue expression predicted response to oral and intrauterine progestogens in endometrial hyperplasia (with and without atypia), such that a greater proportion of responders achieved a reduction in HE4 tissue expression compared to non-responders [10].

The aim of this study was to investigate the expression of HE4 in both serum and endometrial tissue samples taken from patients with AH and stage Ia EEC before and during treatment with LNG-IUS. The objective was to determine whether baseline or changes to serum and tissue HE4 levels during treatment with the LNG-IUS could be used as a predictive biomarker for treatment response.

## 2. Results

### 2.1. Baseline HE4 and Demographics

**Figure 1:**
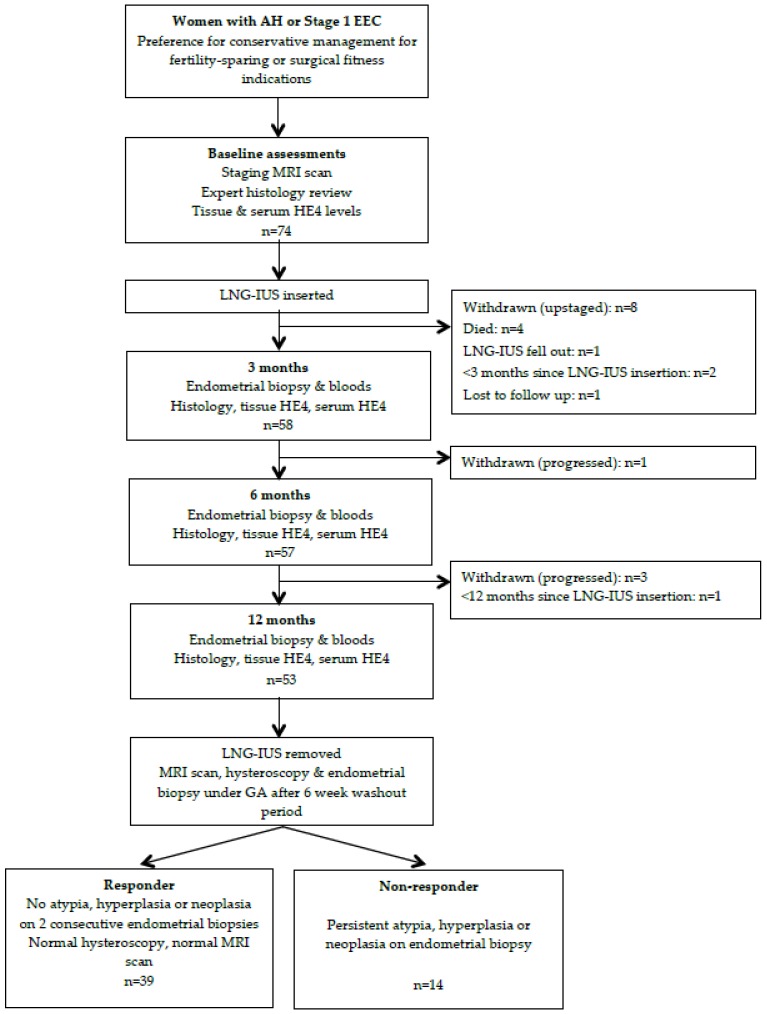
Study flowchart.

Baseline (pre-LNG-IUS insertion) endometrial biopsies and serum samples were obtained from 74 patients. Figure 1 is a study flow chart showing the key steps and reasons for patient withdrawal during the study. Four patients died within 3 months of LNG-IUS insertion due to endometrial cancer (n = 1), myocardial infarction (n = 2) or urinary sepsis following fall with pelvic fracture (n = 1). Three patients did not have a determinable response status at the time of data analysis as two had the LNG-IUS inserted <3 months prior and one <12 months prior. These patients were therefore included in the baseline demographic analyses but not the responder vs. non-responder analyses, although they continued to receive LNG-IUS treatment as per study protocol. All non-responders to the LNG-IUS underwent hysterectomy unless they were medically unfit or declined to do so.

Baseline patient characteristics including age, menopausal status, BMI, smoking status, and diagnosis are shown in Table 1. Eight patients did not have a baseline serum HE4 result available and seven did not have a baseline tissue HE4 available due to sample inadequacy/insufficiency. Baseline serum HE4 correlated positively with age (PCC 0.618, *p* < 0.001, n = 66) and BMI (PCC 0.328, *p* = 0.007, n = 66). Baseline serum HE4 was also higher in post-menopausal compared to pre-menopausal patients (*p* < 0.001) and higher in G1EEC compared to AH (*p* = 0.003) (Table 2). There was no significant difference in baseline serum HE4 in patients with AH vs. G2EEC (*p* = 0.110) or G1EEC vs. G2EEC (*p* = 0.521). Serum HE4 was also not significantly different in smokers compared to non-smokers. (Table 2)

### 2.2. Baseline Characteristics in Responders vs. Non-Responders to LNG-IUS

Of 57 patients for whom response to the LNG-IUS was histologically determinable, 39 (68%) were responders and 18 (32%) non-responders. 83% (25/30) of patients with AH were responders compared to 56% (15/27) of Stage 1a EEC patients. There was no difference in mean age (52.2 ± 2.7 vs. 57.4 ± 3.5 years, *p* = 0.303) or BMI (44.5 ± 1.7 vs. 48.0 ± 3.0 kg/m^2^, *p* = 0.549) in responders compared to non-responders. There was also no significant difference in proportion of pre-menopausal compared to postmenopausal patients who responded to LNG-IUS (74% vs. 65%, *p* = 0.560).

### 2.3. Baseline Serum HE4 in Responders vs. Non-Responders to LNG-IUS

49 out of 57 patients for whom response status was available had a sufficient baseline serum sample available to determine HE4 concentration. Baseline serum HE4 was significantly lower in responders compared to non-responders to the LNG-IUS (62.1 ± 1.1 pM vs. 125.6 ± 1.3 pM, *p* = 0.014) (Table 3). Multinomial logistic regression showed that on average, when considering age, grade, BMI, menopausal status, smoking status and diagnosis (AH vs. Stage 1a EEC) as confounding variables, higher baseline serum HE4 was predictive of resistance to the LNG-IUS (*p* = 0.005), whereas no other variables reached statistical significance. For a 10% increase in serum HE4, the odds ratio of response was 0.792 (95% CI 0.651–0.967, *p* = 0.022, n = 49) i.e., for every 10% increase in baseline serum HE4, the odds of response to the LNG-IUS decreased by 21% (95% CI 3–35%).

**Figure 2:**
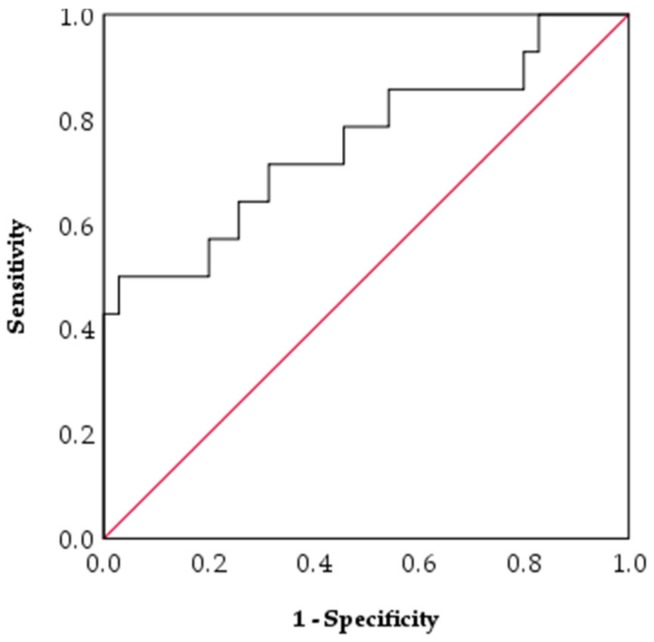
Receiver operating characteristic (ROC) curve analysis of baseline serum HE4 for distinguishing non-responders from responders to the LNG-IUS in AH/ Stage 1a EEC (AUC = 0.755 ± 0.084, 95% CI 0.590–0.921, *p* = 0.006, n = 49).

A receiver operating characteristic (ROC) curve was constructed for baseline serum HE4 and response to LNG-IUS, with an area under curve (AUC) of 0.755 ± 0.084 (95% CI 0.590–0.921, *p* = 0.006, n = 49) (Figure 2). A serum HE4 cut off of 38pM and below had a 100% specificity and 17% sensitivity for predicting a positive response to the LNG-IUS. A serum HE4 cut-off of 165pM and above had 100% specificity and 39% sensitivity for predicting a lack of response to LNG-IUS. 71% (25/35) of responders had a baseline serum HE4 that was below the previously described diagnostic cut-off of 70 pM [26,27] compared to 36% (5/14) of non-responders (*p* = 0.02).

### 2.4. Change in Serum HE4 from Baseline during LNG-IUS Therapy

**Figure 3:**
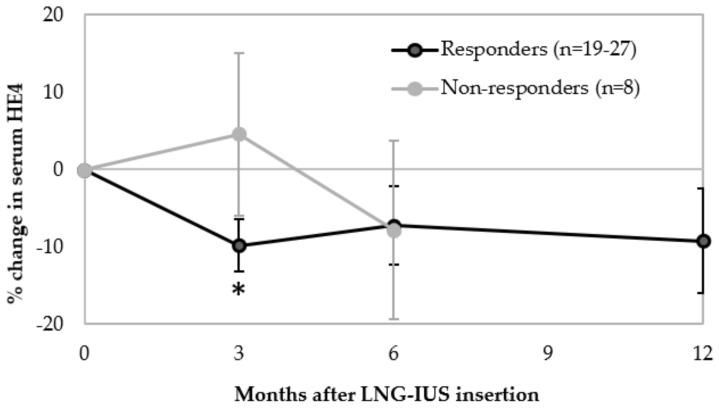
Percentage change in serum HE4 from baseline following LNG-IUS insertion in responders (at 3, 6, and 12 months) compared to non-responders (at 3 and 6 months) (* *p* = 0.008, change from baseline).

35 out of 49 patients in whom response was determinable had sufficient serum samples available at both baseline and at least one of 3, 6 and/or 12 months. Responders to the LNG-IUS had a significant mean reduction in serum HE4 (−9.8 ± 3.4%, 95% CI −16.7 to −2.8%, *p* = 0.008, n = 27)) between baseline and 3 months, whereas non-responders showed no significant change (4.6 ± 10.5%, 95% CI −20.2 to 29.4%, *p* = 0.676, n = 8) (Figure 3). This distinction was lost by 6 months, as responders showed no significant % change in HE4 between baseline and 6 months (*p* = 0.176, n = 25) or 12 months (*p* = 0.184, n = 19). Similarly, non-responders did not display a significant % change in serum HE4 between baseline and 6 months (*p* = 0.522, n = 8) (Figure 3). There was also no significant difference in proportion of responders compared to non-responders who had a reduction in serum HE4 compared to stable/increased HE4 between baseline and 3 months (67% vs. 50% *p* = 0.392) or baseline and 6 months (64% vs. 75%, *p* = 0.565). When comparing the mean % change between baseline and 3 months or 6 months post-LNG-IUS insertion in responders vs. non-responders, there was no significant difference (*p* = 0.227 and *p* = 0.961, respectively). This remained the case when considering % change in BMI as a covariable at 3 months (*p* = 0.895) and 6 months (*p* = 0.417). There were insufficient non-responders who still had the LNG-IUS at 12 months (n = 3) to allow comparison with responders.

### 2.5. Baseline Tissue HE4 Expression, Patient Demographics, and Serum HE4

**Figure 4:**
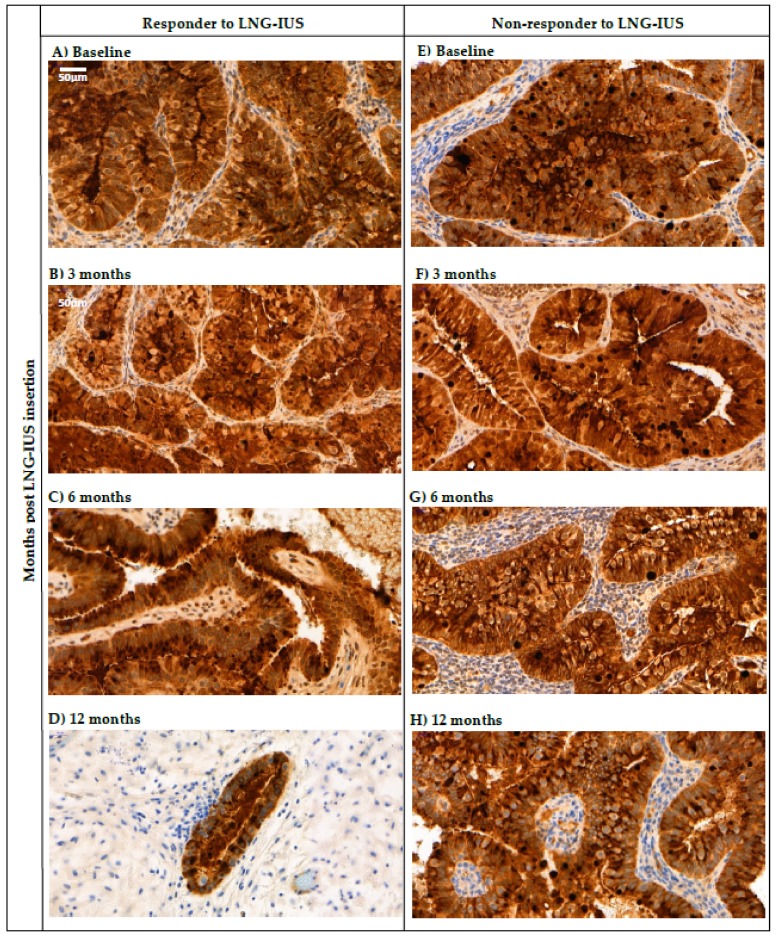
HE4 expression in endometrial biopsies taken from a responder (**A**–**D**) and non-responder (**E**–**H**) to the LNG-IUS with Stage Ia endometrial cancer at baseline and 3, 6, and 12 months post-LNG-IUS insertion. 40× magnification using CaseViewer 3.2 application.

HE4 expression in endometrial tissue biopsies was visible as heterogenous diffuse staining of the cytoplasm of the endometrial glandular epithelium with no staining in adjacent stromal tissue (Figure 4). There was no significant difference in mean age (*p* = 0.466) or BMI (*p* = 0.181) in patients with low, medium or high HE4 tissue expression (Table 4). There was also no significant difference in proportions of low, medium and high HE4 expression in biopsies from patients with AH vs. G1EEC vs. G2EEC (*p* = 0.822) (Table 4) or pre-menopausal vs. post-menopausal patients (*p* = 0.344). 51 out of 57 patients for whom response status was available had a sufficient baseline tissue sample available to determine HE4 expression. There was also no significant difference in HE4 expression in responders vs. non-responders to the LNG-IUS (*p* = 0.999). Mean serum HE4 was not significantly different in patients with low, medium or high HE4 expression (*p* = 0.567) (Table 4).

### 2.6. Changes in Tissue HE4 Expression after LNG-IUS Insertion in Responders vs. Non-Responders

38 out of 49 patients for whom response was determinable had both a baseline tissue sample and at least one of 3-, 6-, and 12-month samples available. There was no significant difference in proportion of patients who had a reduced H-score vs. same or increased H-score in responders compared to non-responders between baseline and 3 months (*p* = 0.280), 6 months (*p* = 0.710), or 12 months (*p* = 0.545) (Table 5). Figure 4 demonstrates persistently strong HE4 expression in the glandular epithelium of biopsies taken from both a responder (Figure 4A–D) and a non-responder (Figure 4E–H) to the LNG-IUS at baseline and at 3, 6, and 12 months post-LNG-IUS insertion.

## 3. Discussion

This study suggests that serum HE4 is an independent predictive biomarker for response to LNG-IUS treatment in patients with AH and Stage Ia EEC. Baseline serum HE4 was significantly lower in responders compared to non-responders to the LNG-IUS, even when correcting for age, menopausal status, BMI, histological grade and smoking status. This supports that lower serum HE4 is predictive of therapy response and higher serum HE4 is predictive of resistance. In this study, all patients with a baseline serum HE4 of 38pM and below responded to the LNG-IUS and conversely, all patients with a baseline serum HE4 of 165pM and above were resistant to treatment. A greater proportion of responders compared to non-responders (71% vs. 36%) had a serum HE4 below the previously described diagnostic cut-off of 70pM [26,27]. Responders to the LNG-IUS showed a significant reduction in serum HE4 of ~10% between baseline and 3 months, whereas there was no significant change in non-responders. This distinction was not present beyond 3 months. When comparing the % change in serum HE4 in responders vs. non-responders, this was not significantly different at 3 or 6 months post-LNG-IUS insertion and there was no significant difference in proportion of responders compared to non-responders who had reduced vs. stable/increased serum HE4 between baseline and 3 or 6 months. This suggests that measuring interval changes in serum HE4 from baseline is not reliable for distinguishing between responders and non-responders to the LNG-IUS.

Since higher serum HE4 is known to be associated with higher stage and grade tumours [24], which are both predictive of increased resistance to LNG-IUS [5,17,20,21,22,23,24], it is logical that non-responders had a higher mean baseline serum HE4 compared to responders. The higher serum HE4 could be attributed to non-responders having larger tumours which produce more HE4 and/or produce it at a faster rate due to higher cell turnover. Equally, extracellular HE4 has been shown to enhance proliferation in endometrial cancer cell lines in vitro ([28,29], so HE4 could be a driver of increased tumour aggressiveness, proliferation and resistance to progestogens rather than simply a product of it. Following these principles, it might be expected that responders to the LNG-IUS should have had a greater reduction in serum HE4 as a result of greater reduction in size and grade of tumours. However, change in serum HE4 between baseline and 3 or 6 months was not significantly different in responders compared to non-responders to the LNG-IUS. This may be because interval changes in HE4 secretion are not directly proportional to changes in tumour size and grade, or because HE4 is expressed in many non-malignant tissues [16]. Also, dynamic changes in production of HE4 by non-malignant organs, including normal endometrium, might have affected the overall changes in serum HE4 between timepoints after LNG-IUS insertion. Additionally, the lack of significant change in serum HE4 from baseline in non-responders during LNG-IUS therapy may be because some non-responders had a partial response and/or developed stabilised disease that resulted in stable serum HE4 levels.

HE4 expression in endometrial tissue biopsies was heterogeneous and visible as strong and diffuse staining in the cytoplasm of glandular epithelium, similar to previous studies [16,20,24]. Baseline tissue HE4 expression was not significantly different in responders compared to non-responders to the LNG-IUS. Changes in HE4 tissue expression between baseline and 3, 6, and 12 months post-LNG-IUS insertion were also not significantly different in responders compared to non-responders. This suggests that neither baseline nor interval changes in tissue HE4 expression during LNG-IUS therapy are predictive of response. In addition, baseline tissue HE4 expression showed no significant association with age, menopausal status, histological grade or BMI. There was also no significant association between baseline serum HE4 and tissue HE4 levels, with mean baseline serum HE4 being similar in patients with low, medium and high baseline HE4 expression. This could be at least partly due to the semi-quantitative H-score method used to measure HE4 staining, which might not be sensitive enough to identify subtle differences in HE4 expression. It could also be due to the heterogenous nature of HE4 expression which means that HE4 expression in biopsies might not be representative of the entire tumour. This supports a recent study that showed that serum HE4 and tissue HE4 expression in paired samples displayed a low concordance, and serum HE4 was recommended as a more precise method of clinically measuring HE4 compared to tissue expression [20]. The fact that changes in tissue HE4 expression from baseline were not predictive of LNG-IUS response or resistance contrasts with a recent study by Orbo et al. 2016 [10]. They showed that a higher proportion of responders to progestogen therapy for hyperplasia (with or without atypia) had reduced tissue HE4 expression between baseline and 6 months compared to non-responders. However, Orbo et al. 2016 did not include any non-responders to the LNG-IUS or any endometrial carcinoma patients, and most patients received oral progestogens. In addition, the majority of their patients had hyperplasia without atypia so it is possible that non-atypical hyperplasia displays a more significant and/or detectable reduction in tissue HE4 expression compared to AH/Stage Ia EEC. In addition, the heterogeneity of tissue HE4 expression in AH and EEC is a further limitation that could limit reproducibility of results between studies.

The strengths of this study are that the design was prospective, and patients were selected consecutively, which minimised bias. In addition, 68% of AH/Stage Ia EEC patients responded to the LNG-IUS by 12 months, with AH showing a higher response rate compared to Stage I EEC, similar to previous studies [7,8,9,10,11] supporting that the sample is representative of the background patient population. Baseline serum HE4 was also positively associated with patient age, BMI, menopause, in agreement with previous studies [17,18,22,23,24,30,31]. Baseline serum HE4 was also significantly higher in G1EEC compared to AH, in support of previous studies [21,22,32]. There was also no significant difference in age or BMI in responders compared to non-responders, minimising confounding bias. Although a previous study reported levels of serum HE4 29% higher in smokers compared to non-smokers [30,33] in healthy patients, in the current study serum HE4 trended higher in smokers but did not reach statistical significance, probably due to only ~10% of patients in this study being smokers. Limitations of the study were the small sample size and smaller proportion of non-responders compared to responders available for comparison. There were also not enough non-responders who had both baseline and 12-month serum samples available to enable comparison of change in serum HE4 between these timepoints in responders vs. non-responders.

Identifying women who are likely to respond to conservative management with the LNG-IUS is a clinical necessity [13]. The incidence of endometrial cancer is rising alongside obesity rates in an ageing population. Elderly women are less likely to receive surgical management at least partly due to poor performance status and/or increased comorbidities [34,35]. At the same time, pre-menopausal women may choose conservative management to preserve their fertility. Advances in pre-operative staging will enable more women with low-risk disease to be accurately identified for non-surgical management [36,37]. Therefore, the proportion of women receiving LNG-IUS therapy for AH/Stage 1a EEC is likely to increase. Whilst response rates in excess of 60% have been reported to the LNG-IUS, many studies also describe women who have progressed on progestogens with serious long-term consequences [5,38,39,40]. A simple blood test that can differentiate between endometrial neoplastic abnormalities that will or will not respond to the LNG-IUS could facilitate informed decision-making and individualised care. Indeed, “can we predict at the time of diagnosis which endometrial cancers and precancerous lesions will respond to hormone treatments” was ranked within the top ten most important unanswered research questions in endometrial cancer according to patients, carers, and healthcare professionals in our recently completed James Lind Alliance Womb Cancer Priority Setting Partnership [41]. This study supports that serum HE4 is a promising biomarker for predicting response to LNG-IUS therapy.

## 4. Materials & Methods

### 4.1. Patients and Sample Collection

Endometrial tissue biopsy and concurrent serum samples were obtained as part of the “MIrena for the Reduction of Endometrial Neoplastic Abnormalities” (MIRENA) study from June 2014–December 2017 at St Mary’s Hospital, Manchester University NHS Foundation Trust, UK. In this study, consecutive patients with histologically and radiologically (staging MRI) confirmed AH or stage Ia EEC who were unsuitable for primary surgery or preferred fertility-sparing management, were selected for treatment with LNG-IUS. Patients were considered unsuitable for surgery if they were clinically assessed to have a high risk of morbidity/mortality due to poor performance status, medical comorbidities and/or obesity, or if they declined surgery. Inclusion criteria were age >18 years, biopsy-proven AH or well-differentiated EEC with <50% myometrial invasion on MRI scan (2009 FIGO Stage 1a). Exclusion criteria were non-endometrioid, mixed histology or concerning pathological features, deep myometrial invasion on imaging (Stage 1b), progestin contra-indicated and/or inability to sample the endometrium pre/post-LNG-IUS. Baseline serum and endometrial biopsies were taken immediately before LNG-IUS (Mirena^®^ levonorgestrel 20 micrograms/24 h) insertion and at 3-month intervals post-insertion. All histology sections were assessed by at least two specialist gynaecological pathologists to confirm tumour grade and histological subtype. At 12 months, the LNG-IUS was removed and a repeat biopsy taken at 6 weeks to confirm histological status. Baseline features were recorded just before LNG-IUS insertion including age, menopausal status and smoking status, and BMI was recorded at every timepoint. Responders to the LNG-IUS were defined as patients with two or more consecutive biopsies showing no cytological atypia, hyperplasia or neoplasia during and/or following LNG-IUS treatment. Samples taken at baseline, 3, 6, and 12 months post-LNG-IUS insertion were used for analysis.

### 4.2. Immunohistochemistry 

Endometrial tissue sections were formalin-fixed, paraffin-embedded, and stored at room temperature until analysis. The blocks were cut to 4 micrometre thickness sections using a microtome and mounted onto glass slides. Following deparaffinisation and rehydration, immunohistochemistry was performed using the Bond Max automated system (Leica Biosystems, Wetzlar, Germany) using an optimised staining protocol. Heat-driven antigen retrieval was carried out at 98 degrees Celsius for 20 min and sections were incubated in primary antibody for 60 min. The primary antibody was the HE4 12A2 monoclonal IgG1 antibody (Fujirebio Diagnostics, Inc., Malvern, PA, USA), provided at an initial concentration of 14.8 mg/mL and diluted to 1/4000 in BOND antibody diluent. Following primary antibody incubation, sections were incubated with polymer for 8 min and developed with mixed-DAB refined solution for 10 min and counterstained with haematoxylin. Positive and negative controls were used in all experiments. The sections were then dehydrated and mounted onto glass slides.

### 4.3. Quantification of HE4 Staining 

The HE4 stained endometrial sections were scanned using the 3DHistech Pannoramic 250 Flash III scanner and images analysed using the CaseViewer 3.2 application. Four representative areas containing endometrial glandular epithelium 475 × 240 micrometres were selected from each section with guidance from a gynaecological histopathologist at ×40 magnification. HE4 immunostaining in the glandular epithelium was evaluated independently by two observers based on the semi-quantitative H-score method [42]. Both observers were blinded to patient demographics, diagnosis and response status. Cytoplasmic staining was graded for intensity (0 = no staining, 1 = weak, 2 = medium, 3 = strong) and percentage of positive cells at each intensity (0 = ≤5%, 1 = 6–20%, 2 = 21–40%, 3 = 41–60%, 4 = 61–80%, 5 = >80%). Each intensity score was multiplied by the corresponding percentage score and these were added to give an H-score for each area ranging from 0–16, as demonstrated by the following formula: H-score = (% of cells with intensity score 1 × 1) + (% of cells with intensity score 2 × 2) + (% of cells with intensity score 3 × 3), where “% of cells” is assigned a value between 0-5 as described above. Any discrepancies of >10% between scores of the two observers for each area were discussed and a consensus reached. The mean scores of the four areas was calculated to give an overall H-score for each tissue section. The H-scores were then categorised as high (H-score = ≥14), medium (H-score = 11–13) or low (H-score = ≤10) based on the tertiles of the H-scores of the baseline samples. 

### 4.4. Quantification of HE4 in Human Serum

Blood was obtained via venepuncture from patients, processed within an hour of acquisition and the serum stored at −80 °C until analysis. The samples were thawed to room temperature and enzyme-linked immunosorbent assay (ELISA) carried out to measure HE4 concentration using the ELISA detection kit provided by Fujirebio (HE4 EIA kit, Catalogue 404-10, Fujirebio Diagnostics AB) using the manufacturer’s protocol, reagents, and controls. ELISA was carried out on paired aliquots, and if there was greater than 10% difference between readings, further aliquots were tested until two concordant values obtained.

### 4.5. Ethical Approval

Ethical approval for this study was provided by the North West Haydock Research Ethics Committee (14/NW/0056) and all patients gave written, informed consent to participate. The study was prospectively registered on the ISRCTN database (31662931).

### 4.6. Statistical Analysis

Data was tabulated in Microsoft Excel and statistical analysis carried out using IBM SPSS Statistics for Windows, Version 23.0. Armonk, NY: IBM Corp. Serum HE4 values were natural log (ln) transformed for all parametric analyses and mean lnHE4 values back transformed to obtain geometric mean HE4 values. Two-sample T-tests were used to determine association of baseline HE4 with grade, menopausal status, smoking status and response to LNG-IUS. Pearson correlation coefficient (PCC) was used to correlate baseline serum HE4 with BMI and age, and all three factors were ln transformed for this analysis. Multinomial logistic regression was carried out to account for confounding variables. Percentage change in HE4 between baseline and 3, 6, and 12 months was compared in responders vs. non-responders using two-sample T-tests. In order to account for changes in HE4 being affected by change in BMI between timepoints, analysis of co-variance (ANCOVA) was carried out. Chi-squared test was carried out to analyse the association between baseline H-score and serum HE4, grade, menopausal status and response to the LNG-IUS. One-way analysis of variance (ANOVA) with post-hoc Bonferroni test was used to compare mean baseline serum HE4, patient age and BMI in low, medium and high H-score histology. Fischer’s test was used to assess the difference in proportion of sections showing stable/ increased vs. decreased HE4 tissue expression or change in serum HE4 in responders compared to non-responders between different timepoints. Mean values are presented ± standard error. A *p* < 0.05 was considered statistically significant.

## 5. Conclusions

This study suggests that baseline serum HE4, but not tissue HE4 expression, is a predictive biomarker for response to LNG-IUS treatment in stage Ia EEC and AH, and higher levels of serum HE4 independently predict resistance to treatment. In contrast, baseline tissue HE4 expression was not predictive of LNG-IUS response and did not correlate with corresponding serum HE4 levels, suggesting tissue HE4 expression is not useful for predicting or monitoring therapy response. Although responders showed an initial reduction in serum HE4 between baseline and 3 months, interval changes in serum HE4 and tissue HE4 expression between baseline, 3, 6, and 12 months were overall not predictive of therapy response and the proportion of patients with reduced or stable/increased serum HE4 was not significantly different in responders compared to non-responders at 3 and 6 months. This suggests that neither changes in serum HE4 nor tissue HE4 are clinically useful for predicting response to the LNG-IUS. Further studies of baseline serum HE4 in larger populations receiving LNG-IUS for AH/Stage I EEC would be useful to confirm the results of this study and determine the most appropriate serum HE4 cut-offs for predicting therapy response and/or resistance. As there is a positive association between age and baseline HE4, age-adjusted rather than a single cut-off HE4 level is likely to have more clinical utility for predicting response. Further studies of the function of HE4 at a molecular and cellular level would be useful to determine the mechanism of HE4 production, function, and progestogen resistance in AH and EC.

## Figures and Tables

**Table 1:** Baseline patient characteristics (n = 74).

Age (Years)	
Mean	54.4 ± 2.0
Range	25–89
**Menopausal Status**	
Pre-menopausal	28 (38%)
Post-menopausal	46 (62%)
**Body Mass Index (kg/m2)**	
Mean	44.8 ± 1.3
Range	16.4–70.1
**Diagnosis**	
AH	34 (46%)
G1EEC	35 (47%)
G2EEC	5 (7%)
**Smoking Status**	
Smoker	7 (9%)
Non-smoker	67 (91%)

**Table 2:** Mean baseline serum human epididymis protein 4 (HE4) according to histology, menopause and smoking status (n = 66) (* *p* < 0.05).

Baseline Characteristics	Baseline Serum HE4 (pM)	95% CI	N	*p*-Value
**AH**	58.7 ± 1.1 *	49.0–70.2	32	* AH vs. G1EEC *p* = 0.003 AH vs. G2EEC *p* = 0.110 G1EEC vs. G2EEC *p* = 0.521
**G1EEC**	105.8 ± 1.1 *	75.6–147.9	31	
**G2EEC**	90.0 ± 1.2	42.7–189.7	3	
**Pre-Menopausal**	46.9 ± 1.1	36.2–60.6	25	*p* < 0.001
**Post-Menopausal**	108.4 ± 1.1	87.5–134.1	41	
**Smoker**	106.3 ± 1.3	51.1–221.1	6	*p* = 0.317
**Non-Smoker**	76.6 ± 1.1	62.7–93.5	60	

**Table 3:** Mean baseline serum HE4 in responders compared to non-responders to the levonorgestrel-releasing intrauterine system (LNG-IUS) in patients with atypical hyperplasia (AH)/Stage1a low grade endometrial cancer (EEC).

Response to LNG-IUS	Baseline Serum HE4 (pM)	95% CI	N	*p*-Value
**Responders**	62.1 ± 1.1	52.7–73.2	35	0.014
**Non-Responders**	125.6 ± 1.3	74.5–211.7	14	

**Table 4:** Mean baseline age, body mass index (BMI) and serum HE4 in biopsies with low, medium and high HE4 expression, and proportion of patients with low, medium and high baseline HE4 expression according to grade, menopausal status and response to the LNG-IUS. 95% confidence interval (CI) in brackets.

Baseline Characteristics and Response to LNG-IUS	Baseline HE4 Expression				
	Low	Medium	High	N	*p*-Value
**Age (years)**	51.8 ± 3.3 (45.0–58.6) n = 30	57.1 ± 3.1 (50.8–63.4) n = 26	51.5 ± 5.7 (38.8–64.1) n = 11	67	0.466
**BMI (m/kg^2^)**	41.3 ± 2.5 (36.2–46.3) n = 30	45.7 ± 1.7 (42.1–49.3) n = 26	48.0 ± 3.4 (40.3–55.6) n = 11	67	0.181
**Serum HE4 (pM)**	69.1 ± 1.2 (50.0–96.7) n = 24	88.5 ± 1.9 (62.0–126.4) n = 24	85.2 ± 1.3 (48.9–148.6) n = 11	59	0.567
**AH**	12 (39%)	13 (42%)	6 (19%)	31	0.822
**G1EEC**	15 (47%)	12 (38%)	5 (16%)	32	
**G2EEC**	3 (75%)	1 (25%)	0 (0%)	4	
**Pre-Menopausal**	7 (27%)	15 (58%)	4 (15%)	26	0.666
**Post-Menopausal**	15 (37%)	19 (46%)	7 (17%)	41	
**Responder to LNG-IUS**	15 (44%)	15 (44%)	4 (12%)	34	0.999
**Non-Responder to LNG-IUS**	8 (47%)	7 (41%)	2 (12%)	17	

**Table 5:** Proportion of responders vs. non-responders to the LNG-IUS showing reduced vs. same/increased H score compared to baseline after LNG-IUS insertion.

Time Post-LNG-IUS Insertion	Response to LNG-IUS	Reduced H Score	Same/Increased H Score	N	*p*-Value
3 Months	Responder	7 (30%)	16 (70%)	23	0.280
	Non-responder	2 (15%)	11 (85%)	13	
6 Months	Responder	7 (33%)	14 (67%)	21	0.710
	Non-responder	3 (25%)	9 (75%)	12	
12 Months	Responder	4 (20%)	16 (80%)	20	0.545
	Non-responder	0 (0%)	7 (100%)	7	
